# Hospitals

**Published:** 1876-05

**Authors:** 


### Hospitals.

### COOK COUNTY HOSPITAL.

Service Prof. J. P. Ross.

(Reported by D. A. K. Steele, M.D.)

Case 1. *Progressive Locomotor Ataxia.*—W. H. S., æt. 26, bricklayer, American; admitted Sept. 15, 1875. Patient was in the army as drummer for twenty-one months. For six months afterwards he was employed as a government teamster. In both situations was constantly exposed to changes of temperature and frequent wettings. He drank occasionally, enjoyed pretty good health until about six years ago, when one evening he suddenly became very faint and dizzy and was obliged to go to bed. During this attack, which lasted about twenty minutes, he had no pain but a great difficulty in breathing. Slept all night and was able to work the following day. With the exception of an attack of intermittent fever which continued more or less severely for one year, he was well until about sixteen months after the first attack, when one evening while walking the streets he again became weak and dizzy and was obliged to sit down. Carried home he was ill during the next two months, seized with the same sensations whenever he attempted to walk, while he felt quite well when sitting down. Appetite was good. At this time he also noticed that he could not walk well in the dark, that he would get dizzy on stooping over, would fall as soon as he shut his eyes. Ever since this time (for the past three years) he has suffered from this difficulty in walking in the dark or with his eyes shut, and has been subject to attacks of faintness and vertigo, has occasional cramps of the muscles of the calf of leg.

Patient well nourished ; pulse, 54 ; resp. 24 ; appetite good ; bowels constipated. Is unable to stand with eyes closed and heels approximated, immediately staggers and would fall if not supported. Is unable to walk with eyes shut, and has to watch his movements carefully when walking with open eyes ; sensations good in the limbs ; arms seem to be but little affected ; can touch any part of the body with the fingers when the eyes are closed. After a brief treatment with fluid extr. ergot the patient left the hospital Oct. 5.

Case II. *Cancer of Pylorus.*—E. M., æt. 40, Irish laborer ; admitted Oct. 13, 1875. Was perfectly well until last February, when he was attacked with vomiting and severe pain in the stomach ; pain was lancinating in character, and has continued, more or less, ever since. Could not retain his food ; has at times diarrhœa ; has lost seventy-five pounds in weight, and is very much emaciated; tongue furred; pulse rapid and feeble; has severe pain in stomach, cries out as if in great agony; frequent vomiting. Lungs and heart normal; can not find any tumor in hypogastrium by palpation. Patient evidently was failing rapidly, and he died Oct. 15.

Autopsy twelve hours after death. Find a mass of schirrhous deposit at pylorus and in tissues about it. Secondary cancerous deposit in liver; other organs healthy.

CASE III. *Cirrhus—Dropsy.*—C. M., æt. 36, laborer, German. Patient was well until about six weeks ago when he began to be troubled with shortness of breath when he walked fast; occasional pain in the left chest and shoulder; appetite good; bowels regular.

Three weeks ago his feet, legs and abdomen began to swell, and have continued swollen ever since. On admission well nourished. Skin normal; tongue large, flabby, and slightly furred; urine high colored; feet and legs œdematous; abdomen contains fluid.

Physical examination reveals first sound of heart almost purely valvular, no heart murmurs; no albumen in urine; liver irregular.

Ordered ℞. Ext. Colocynth Co. gr.ij.; Potass. Bitart. ʒj. Ter die.

24th. ℞. Quinia Sulph. gr. ij.; Strychnia Sulph. gr. $\frac{1}{32}$; Tr. Ferri. Chlor. gtts. xv. Ter die.

Oct. 19th. Patient leaves hospital much relieved; scarcely any dropsy left; only slight œdema of the feet and ankles during the day.

---

### CENTRAL HOSPITAL FOR THE INSANE, JACKSONVILLE, ILL.

*Case of Peritonitis from Perforation of the Stomach by Foreign Body—Death —Post-mortem.*

(Reported by F. C. WINSLOW, M.D.)

F— F—, male, æt. 26; insane for a year, and during the six months of his residence here was filled with delusions—constantly seeing and conversing with ghosts, and more recently manifesting suicidal propensities.

March 6th. He was visited by friends, and although it was remarked that he was not quite as well as usual, yet he was dressed and around the ward in his ordinary manner.

7th. In the morning was too sick to leave his bed, and on examination was found lying on his back with the knees drawn up, abdomen very tender and slightly tympanitic, diaphragm fixed, breathing chiefly thoracic. The face was flushed, skin hot, tongue dry, brown and tremulous; pulse 120 and wiry in character. He had vomited a little greenish matter in the morning, and refused all ingesta except cold water. The case was pronounced peritonitis, and strong suspicions were entertained that he had swallowed some foreign substance, as a piece of glass or metal.

Without pausing to detail the history, it is sufficient to say that the case presented from day to day an aggravation of the symptoms, and terminated fatally on the 12th.

Post-mortem four hours after death; *rigor mortis* beginning; abdomen tense, but not enormously distended; an incision was made extending from the ensiform appendix to the pubes, and the structures carefully divided down to the peritoneum. On opening the peritoneal cavity a small quantity of serous fluid containing flakes of lymph was found. Both the abdominal and visceral peritoneum—the latter particularly—were found highly congested and inflamed. The stomach contained a little greenish fluid, while the small intestine was almost empty. On passing the hand behind the stomach to remove it, the operator felt a sharp body and withdrew an ordinary sewing needle, the point of which protruding through the gastric walls he had perceived. The intestines were removed entire, and evidences of severe inflammation throughout the small intestine were everywhere present. A condition of positive softening was observed in one portion about ten or twelve inches long, this particular portion of the intestine marking the location of another foreign body—a piece of knitting needle over two inches long. The large intestine was filled with thin yellowish fecal matter.

The needle was found in the stomach, and it is supposed the irritation caused by the point against the diaphragm increased the pain of the respiratory movements always observed in peritonitis.

